# Mitochondrial respiratory chain dysfunction modulates metalloproteases -1, -3 and -13 in human normal chondrocytes in culture

**DOI:** 10.1186/1471-2474-14-235

**Published:** 2013-08-09

**Authors:** Berta Cillero-Pastor, Ignacio Rego-Pérez, Natividad Oreiro, Carlos Fernandez-Lopez, Francisco J Blanco

**Affiliations:** 1 Rheumatology Division, INIBIC-Hospital Universitario A Coruña, A Coruña, Spain

## Abstract

**Background:**

Mitochondrion has an important role in the osteoarthritis (OA) pathology. We have previously demonstrated that the alteration of the mitochondrial respiratory chain (MRC) contributes to the inflammatory response of the chondrocyte. However its implication in the process of cartilage destruction is not well understood yet. In this study we have investigated the relationship between the MRC dysfunction and the regulation of metalloproteases (MMPs) in human normal chondrocytes in culture.

**Methods:**

Human normal chondrocytes were isolated from human knees obtained form autopsies of donors without previous history of rheumatic disease. Rotenone, 3-Nitropropionic acid (NPA), Antimycin A (AA), Sodium azide and Oligomycin were used to inhibit the activity of the mitochondrial complexes I, II, III, IV and V respectively. The mRNA expression of MMPs -1, -3 and -13 was studied by real time PCR. The intracellular presence of MMP proteins was evaluated by western blot. The liberation of these proteins to the extracellular media was evaluated by ELISA. The presence of proteoglycans in tissue was performed with tolouidin blue and safranin/fast green. Immunohistochemistry was used for evaluating MMPs on tissue.

**Results:**

Firstly, cells were treated with the inhibitors of the MRC for 24 hours and mRNA expression was evaluated. An up regulation of MMP-1 and -3 mRNA levels was observed after the treatment with Oligomycin 5 and 100 μg/ml (inhibitor of the complex V) for 24 hours. MMP-13 mRNA expression was reduced after the incubation with AA 20 and 60 μg/ml (inhibitor of complex III) and Oligomycin. Results were validated at protein level observing an increase in the intracellular levels of MMP-1 and -3 after Oligomycin 25 μg/ml stimulation [(15.20±8.46 and 4.59±1.83 vs. basal=1, respectively (n=4; *P<0.05)]. However, AA and Oligomycin reduced the intracellular levels of the MMP-13 protein (0.70±0.16 and 0.3±0.24, respectively vs. basal=1). In order to know whether the MRC dysfunction had an effect on the liberation of MMPs, their levels were evaluated in the supernatants. After 36 hours of stimulation, values were: MMP-1=18.06±10.35 with Oligomycin 25 μg/ml vs. basal=1, and MMP-3=8.49±4.32 with Oligomycin 5 μg/ml vs. basal=1 (n=5; *P<0.05). MMP-13 levels in the supernatants were reduced after AA 60 μg/ml treatment (0.50±0.13 vs. basal=1) and Oligomycin 25 μg/ml (0.41±0.14 vs. basal=1); (n=5; *P<0.05). The treatment of explants with Oligomycin, showed an increase in the positivity of MMP-1 and -3. Explants stimulated with AA or Oligomycin revealed a decrease in MMP-13 expression. Proteoglycan staining demonstrated a reduction of proteoglycan levels in the tissues treated with Oligomycin.

**Conclusions:**

These results reveal that MRC dysfunction modulates the MMPs expression in human normal chondrocytes demonstrating its role in the regulation of the cartilage destruction.

## Background

Osteoarthritis (OA) is a pathology characterized by the destruction of the cartilage and joint dysfunction [1,2]. The cartilage has been always described as an avascular and hypoxic tissue. This is the reason why mitochondria did not have been extensively studied in this tissue. However, our group has previously described that the activity of the mitochondrial respiratory chain (MRC) complexes II and III is reduced in human OA chondrocytes in culture compared to healthy chondrocytes [3,4]. In addition, some haplogroups that codify for genes related to the MRC, confer a higher predisposition to develop the OA disease [5]. In addition, some proinflammatory factors, like cytokines IL-1β and TNF-α, produce a decrease in the activity of the MRC complex I [6]. Among other substances produced by the tissues in the OA joint, nitric oxide (NO) has an important role on the mitochondrial activity [7,8]. Our group described that the NO donor sodium nitroprusside (SNP), reduced the activity of the MRC complex IV [9]. Other groups have related the dysfunction of the MRC complex I to the NO production [10]. In previous studies, our group has also demonstrated that the MRC dysfunction could generate an inflammatory response in the chondrocyte with upregulation of COX-2 and PGE2 production [11-13].

The progressive degradation of the extracellular matrix (ECM) in the tissues like cartilage, bone and synovial tissue is one of the most common events in the rheumatic pathologies [14]. Despite a high number of proteases contribute to the tissue destruction, the family of metalloproteases (MMPs) plays an important role. The MMPs are endopeptidase zinc and calcium dependent enzymes, secreted by resident cells in the tissues as well as by invading cells. Their function is to remodel the ECM in physiological processes (embryogenesis, cellular migration, angiogenesis) and in pathological processes like (tumours, rheumatic pathologies, cardiovascular diseases) [15]. Collagenase 1 or MMP-1, was the first MMP that was described. It is widely expressed in connective tissues like fibroblasts, chondrocytes, monocytes, macrophages and oncogenic cells. It breaks collagen III but also collagen I, II, VII, VIII, X and gelatine. It activates the pro MMP-2 and -9. MMP-13 or collagenase-3 degrades collagen II and collagen I, III, IV, IX, X, XIV, gelatine, laminin, aggrecane and fibronectin [16]. Stromelisin 1 or MMP-3 is expressed in fibroblasts, osteoblasts, osteblasts and chondrocytes. Its targets are collagen II, IV, IX, X, XI and gelatine, as well as elastine, caseine, laminin, fibrinogen and aggrecan. It also activates other procollagenases (-1, -8, -13) and pro MMP-9 [17]. It is highly expressed in late OA phases. There is a direct relation between MMP-3 levels and the OA severity grade [18,19]. Some studies have demonstrated how hyaluronic acid reduces the MMP-3 expression and the cartilage destruction [20-22]. Considering the importance of MMPs in the integrity of the cartilage, and given the role of the mitochondria in the chondrocyte physiology, we evaluated the modulation of MMPs after mitochondrial dysfunction.

## Methods

### Cell culture and experimental conditions

#### ***Cartilage procurement and processing***

Normal human knee cartilage from donors (with ages ranging from 18 to 65 years) with no history of joint disease was provided by the Tissue Bank and the Autopsy Service at CHU de La Coruña. Cartilage slices were removed from the condyles and treated with trypsin 0.5 mg/ml (Sigma-Aldrich, St. Louis, MO) for 15 min at 37°C. Then, the cartilage was incubated overnight in an orbital shaker at 37°C with 2 mg/ml clostridial collagenase (Sigma-Aldrich) in Dulbecco’s modified Eagle’s medium (DMEM; Gibco Life Technologies, Paisley, UK). The cells were resuspended in fetal calf serum (FCS)–enriched DMEM and used in the first passage. For tissue studies, pieces of cartilage 6 mm in diameter and 4 mm in height were cut from cartilage and stimulated in DMEM. The local ethics committee in Galicia, Spain, approved this study.

### mRNA expression studies

mRNA of 5×10^5^ cells per condition, was isolated with Trizol reagent (Invitrogen, Paisley, Scotland, UK), treated with Deoxyribonuclease I amplification grade (Invitrogen) and amplified with a Transcriptor First Strand cDNA Synthesis commercial kit (Roche Diagnostics,). PCR analyses for MMPs and the housekeeping gene hipoxantine phosphoribosiltransferase 1 (HPRT1) were conducted with the LightCycler 4800 SYBR Green I Master kit using the Real Time Light Cycler (Roche Diagnostics). The primers employed were: MMP-1: 5′-gctaacctttgatgctataactacga-3′ (sense) and 5′-tttgtgcgcatgtagaatctg-3′ (antisense); MMP-3: 5′-caaaacatatttctttgtagaggacaa-3′ (sense) and 5′-ttcagctatttgcttgggaaa-3′ (antisense); MMP-13: 5′-ccagtctccgaggagaaaca-3′ (sense) and 5′-aaaaacagctccgcatcaac-3′(antisense); HPRT1: 5′-tgaccttgatttattttgcatacc-3′(sense) and 5′-cgagcaagacgttcagtcct-3′(antisense).

PCR data were analyzed using Relative Expression Software Tool (REST) (Qiagen, Valencia, CA) software, which provides statistical information for comparing groups taking into account issues of reaction efficiency and reference gene normalization.

#### ***Western blot***

After stimulation, cells (5×10^5^ per well) were lysed in 0.2 M Tris–HCl (pH 6.8) containing 2% SDS, 20% glycerol, 1 μg/ml cocktail inhibitor and 1 mM PMSF. Protein concentrations were determined using the BCA reagent assay (Pierce Chemical Co., Rockford, IL, USA). Protein extract (30 μg) was resolved on 12.5% SDS–polyacrylamide gels and transferred to polyvinylidene difluoride membranes (Immobilon P; Millipore, Bedford, MA). These membranes were first blocked for 1 hour at room temperature in Tris buffered saline, pH 7.4, containing 0.1%Tween 20 (TBST) and 5% non fat dry milk, and then incubated overnight at 4°C with anti-MMP-1 human rabbit 1:50 (NeoMarkers, Fremont, California USA), anti-MMP-3 human mouse 1:200 (Chemicon, Temecula, CA, USA) and anti-MMP-13 1:50 human rabbit (Neomarkers) antibody in fresh blocking solution. After thorough washing with TBST, immunoreactive bands were detected by chemiluminescence using corresponding horseradish peroxidase–conjugated secondary antibodies (1:2000, GE Healthcare), enhanced chemiluminescence detection reagents (GE Healthcare), and a LAS 3000 image analyzer. Quantitative changes in band intensities were evaluated with ImageQuant 5.2 software (GE Healthcare). To assure that equal amounts of the total proteins were evaluated, membranes were also hybridized with monoclonal anti-human α-tubulin antibodies (1:2500; Sigma-Aldrich).

#### ***Liberation of MMPs***

The liberation of MMPs to the cell media was evaluated by ELISA according to manufacter instructions. 4.5×10^6^ cells per condition were used in 12 well plates. After 36 hours of stimulation, media was collected and centrifugated at 800 rpm for 5 min. MMP-1 was studied by ELISA with a detection range of 6.25-100 ng/mL (GeHealthcare). For the MMP-3 study (R&D Systems, Abingdon, UK) samples were diluted 1:1000. The detection rate was 0.156-10 ng/mL. The MMP-13 study was performed with a detection range of 0.094–3 ng/ml (GeHealthcare).

#### ***MMPs detection in tissue***

Tissue sections of 6 mm of diameter were stimulated in 48 well plates and frozen in OCT. Slices of 4 μm were fixed in acetone for 1 min at 4°C. Anti-MMP-1 (1:100), anti-MMP-3 (1:50) or anti-MMP-13 (1:50) antibodies were used for 1 h. After three washing steps with PBS anti-rabbit or anti-mouse (1:20) antibodies labelled with peroxidase were used (Dako) for 30 min at room temperature. After washing, 3,3′-diaminobenzidina (DAB) (Dako) was applied for 5 min. After dehydratation in alcohols and mounting *DePeX* (VWR, Bridgeport, NJ, USA), tissues were observed in the microscope.

#### ***Proteoglycan studies***

The levels of proteoglycans were evaluated with toluidin blue in cartilage slices. After fixing in acetone, tissues were dipped in toluidin blue 0.2% (Sigma) in sodium acetate buffer 98 mM, acetic acid 5M pH 4.2, for 20 min. After a washing step with water, samples were dehydrated with alcohol solutions (70°C, 96°C, 100°C). After washing with xylene, *DePeX* was used for mounting and visualization in the microscope with a Nikon camera (Nikon Instruments, Melville, NY). Safranine fast green was also used for proteoglycan detection. For this technique, FFPE tissues were cut in the microtom and washed to get rid of the paraffin. Fast green stained the background for 5 min. After washing for 10 sec in acetic acid and safranin 0.1% for 5 min tissues were dehydrated and mounted. Proteoglycan quantitation was done with Analisys software obtaining relative values.

#### ***Statistical analyses***

The data are expressed as mean ± SE. Individual donor assays were duplicated. The statistical software program SPSS (version 15.0, SPSS, Chicago, IL, USA) was used to perform analysis of variance (ANOVA) and Tukey tests. Differences were considered to be statistically significant at P≤0.05.

## Results

### Intracellular MMP-1, MMP-3 and MMP-13 expression after MRC dysfunction

**Figure 1:**
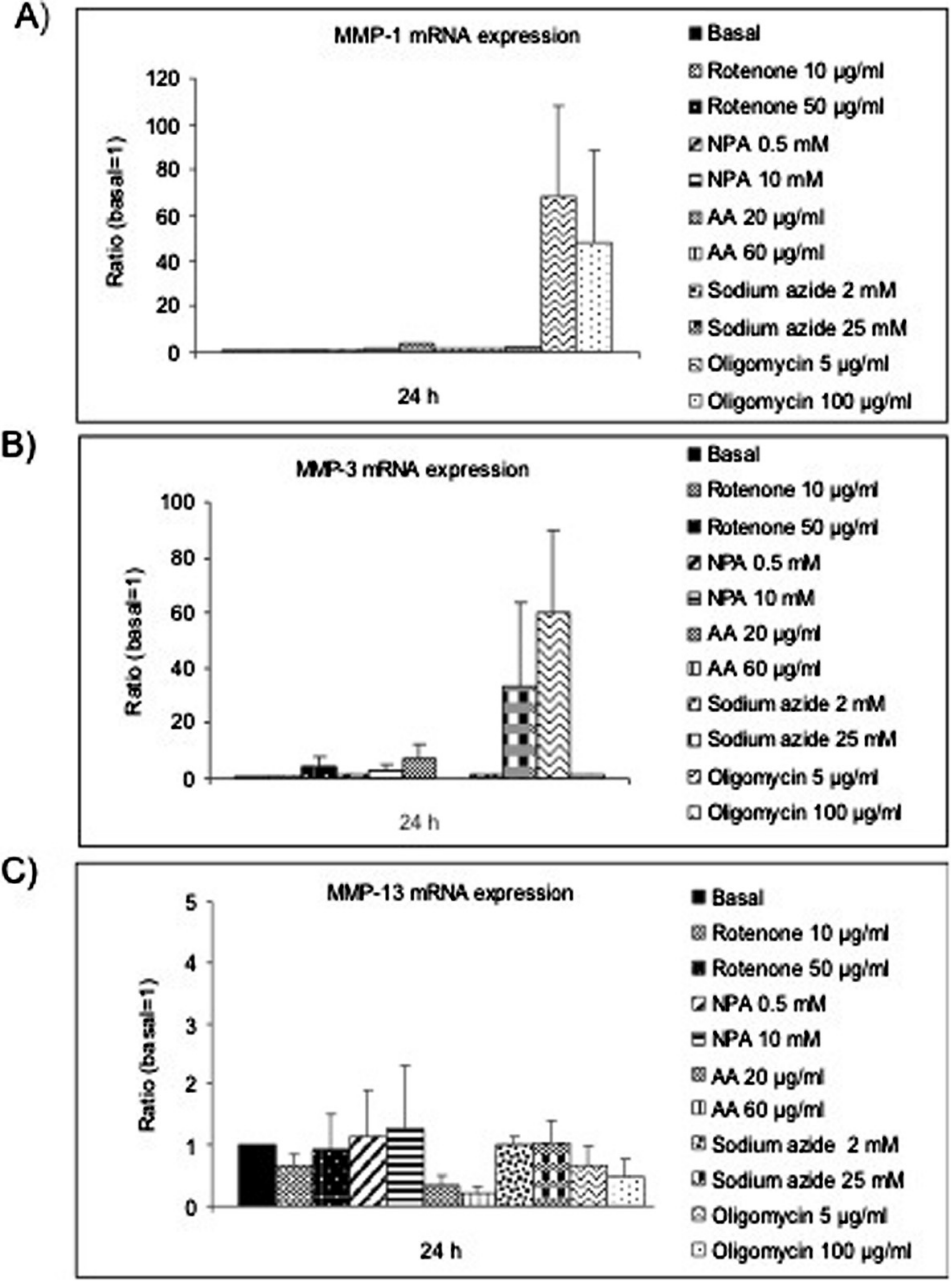
**mRNA expression of MMPs-1, -3 and -13 in chondrocytes after MRC dysfunction.** Chondrocytes were cultured in 6 well plates in basal conditions or with Rotenone (10 and 50 μg/mL), NPA (0.5 and 10 mM), AA (20 and 60 μg/mL), Sodium azide (2 and 25 mM) or Oligomycin (5 and 100 μg/mL) for 24 h. The mRNA was purified and PCR was conducted in order to analyze MMP-1 **(A)**, MMP-3 **(B)** and MMP-13 expression **(C)**. Data are represented as mean ± SE of 9 independent experiments in duplo.

**Figure 2:**
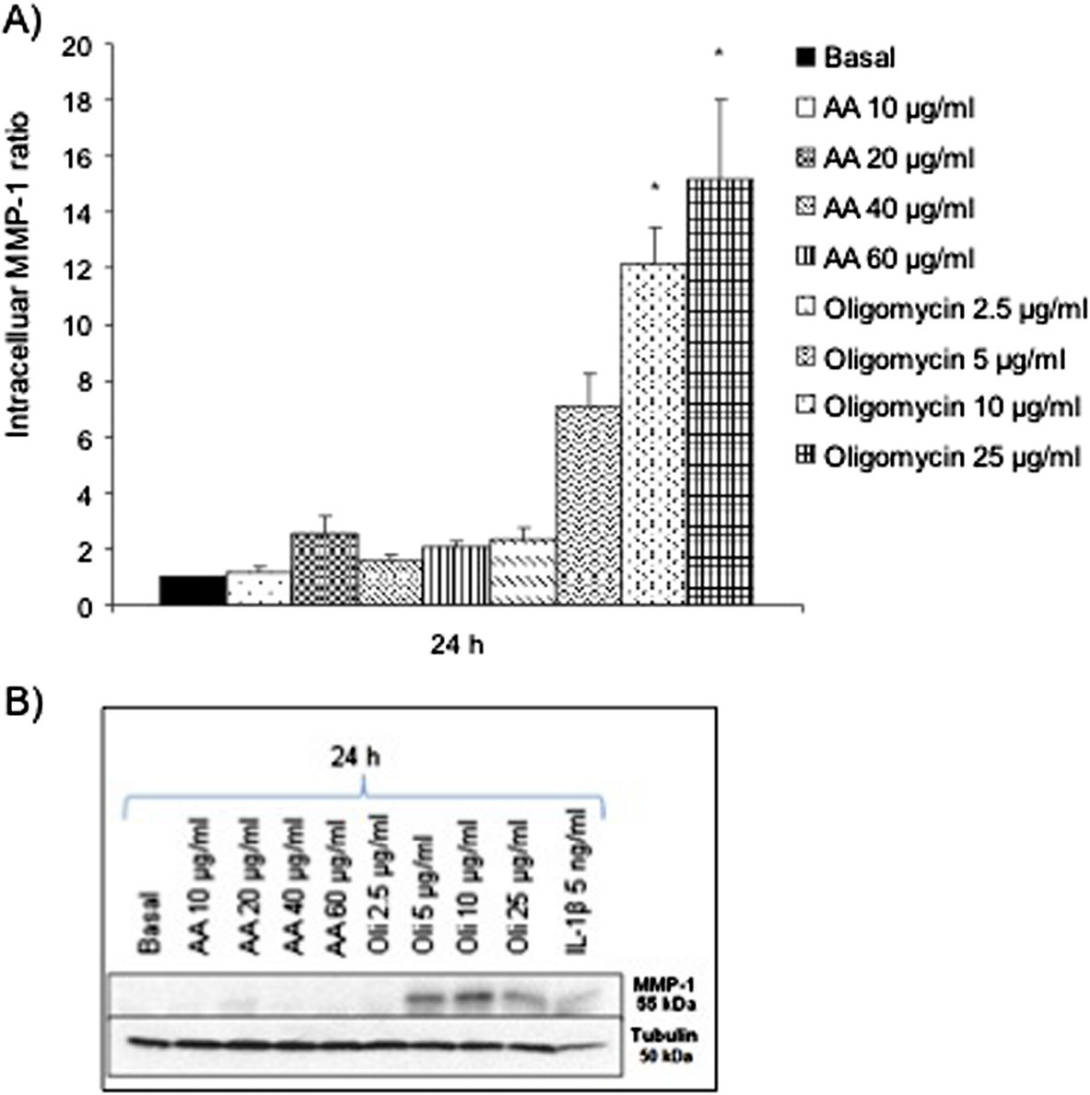
**MMP-1 intracellular protein levels in chondrocytes after MRC dysfunction. A)** Human chondrocytes were cultured in 6 well plates, in basal conditions with AA or Oligomycin for 24 h and intracellular proteins were detected by *western blot*. Data were expressed as a ratio (basal=1) represented as mean ± SE of 4 independent experiments (*P<0.05). **B)** Example of a representative experiment.

**Figure 3:**
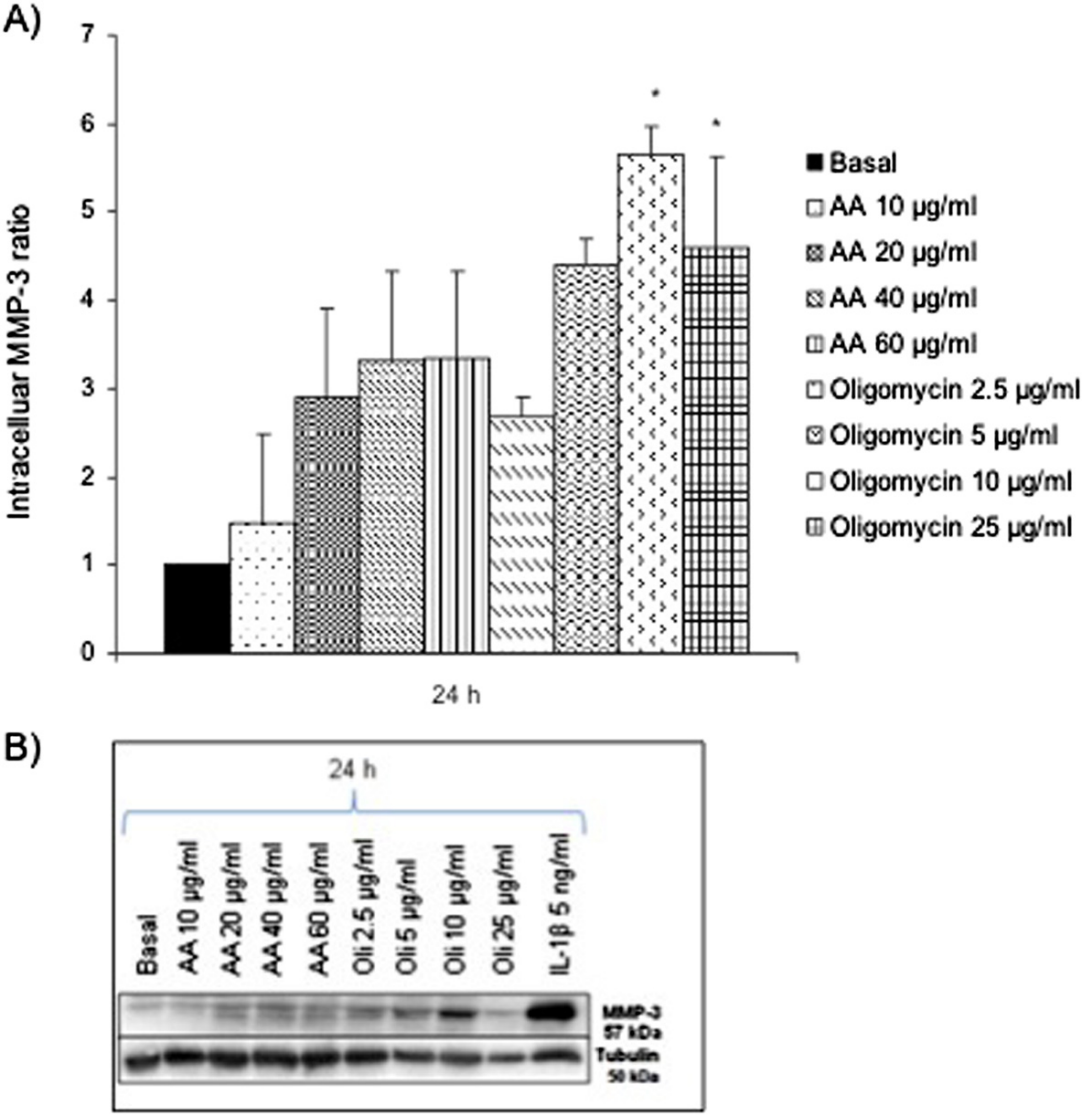
**MMP-3 protein levels in chondrocytes after MRC dysfunction. A)** Human chondrocytes were cultured in 6 well plates in basal conditions, with AA or Oligomycin for 24 h and intracellular proteins were detected by *western blot*. Data were expressed as ratio (basal=1) representing the mean ± SE of 4 independent experiments (*P<0.05). **B)** Example of a representative experiment.

**Figure 4:**
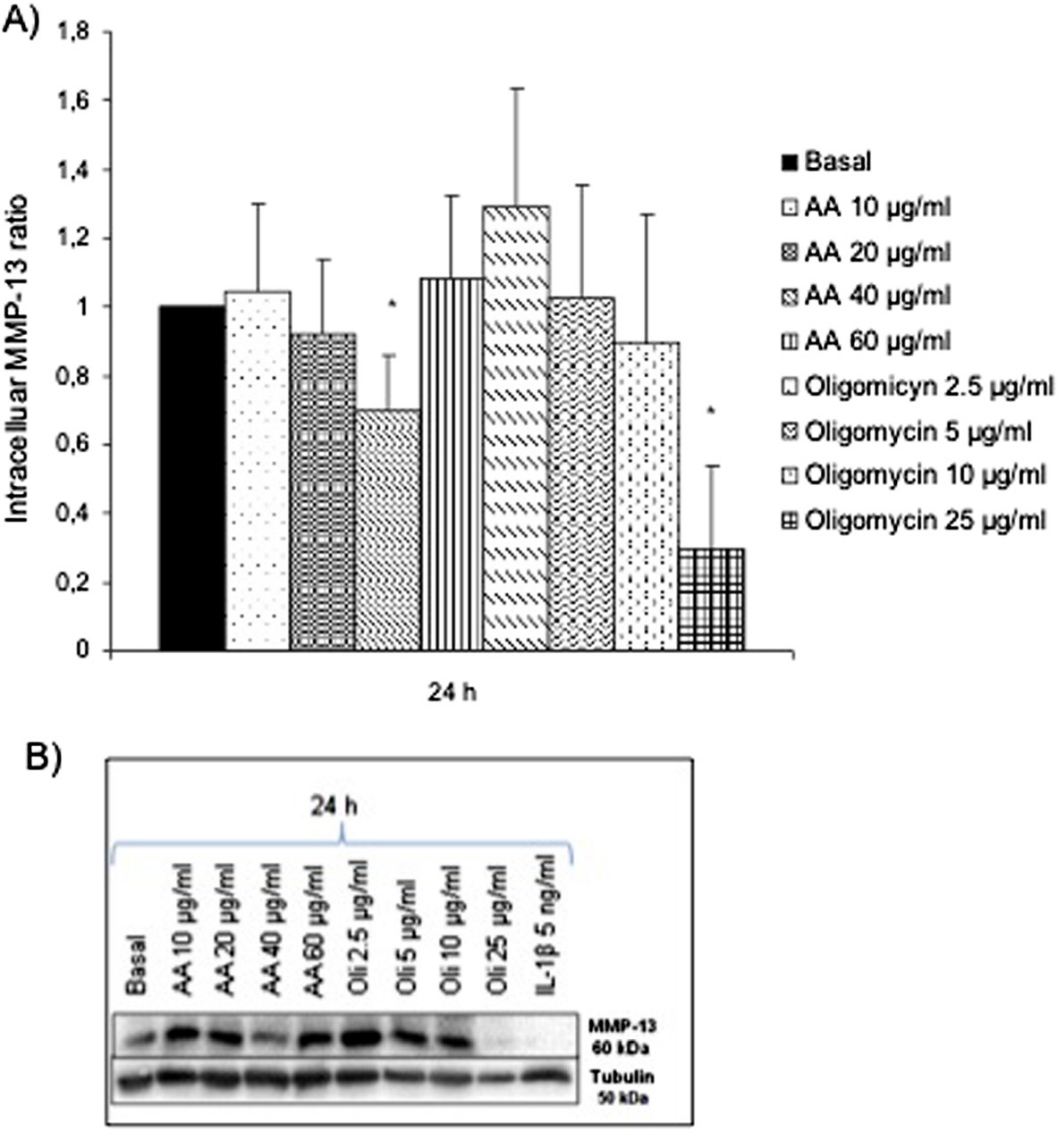
**MMP-13 protein levels in chondrocytes after MRC dysfunction. A)** Human chondrocytes were cultured in 6 well plates in basal conditions, with AA or Oligomycin for 24 h and intracellular proteins were detected by *western blot*. Data were expressed as ratio (basal=1) representing the mean ± SE of 4 independent experiments (*P <0.05).** B)** Example of a representative experiment.

We evaluated the possible modulation at mRNA level of MMPs -1, -3 and -13 after the induction of the MRC dysfunction. According to the bibliography, we used Rotenone 10 and 50 μg/ml to inhibit the MRC complex I, NPA 0.5 and 10 mM to inhibit the MRC complex II, Antimycin A (AA) 20 and 60 μg/ml to inhibit the complex III, Sodium azide 2 and 25 mM to inhibit the complex IV and Oligomycin 5 and 100 μg/ml to inhibit the activity of the complex V. After 24 hours of treatment, we analyzed the mRNA expression of MMPs -1, -3 and -13 as Figure 1 shows. Oligomycin 5 μg/ml produced a tendency in the increase of MMP-1 and -3 expression (Figure 1A, 1B) to 68.10±39.9 and 60.13±29.7 vs. basal=1, respectively (n=9). On the other hand, the inhibition of the complex III with AA 20 μg/ml, produced a decrease in the MMP-13 mRNA expression to 0.34±0.2 vs. basal=1 (Figure 1C). To confirm these results at protein level, we evaluated the intracellular protein expression of these MMPs by western blot (Figures 2, 3 and 4). We stimulated the cells at different concentrations of AA or Oligomycin according to the preliminary mRNA results. The positive control used was IL-1β 5 ng/ml. The treatment of chondrocytes with the inhibitor of complex V (Oligomycin 2.5, 5, 10 and 25 μg/ml) after 24 hours produced an increase in the MMP-1 levels (Figure 2A). The levels increased significantly up to 12.20±3.24 and 15.20±8.46 vs. basal=1, Oligomycin 10 and 25 μg/ml respectively, (n=4; *P<0.05). Figure 2B represents an experiment of 4. As we expected, AA did not induce the MMP-1 modulation according to the mRNA results. In a similar way, MMP-3 was only induced by Oligomycin. Figure 3A shows these levels: at 24 h 5.65±2.08 and 4.59±1.83 vs. basal=1 for the concentrations of 10 and 25 μg/ml, respectively (n=4; *P<0.05). Figure 3B represents an experiment of 4. As we expected, AA did not induce the modulation of MMP-1. MMP-13 decreased after treatment with AA 40 μg/ml and Oligomycin 25 μg/ml (0.70±0.16 and 0.3±0.24 vs. basal=1; n=4; *P<0.05) (Figure 4A and 4B).

### MMPs-1, -3 and -13 liberation in chondrocytes after the induction of the MRC dysfunction

**Figure 5:**
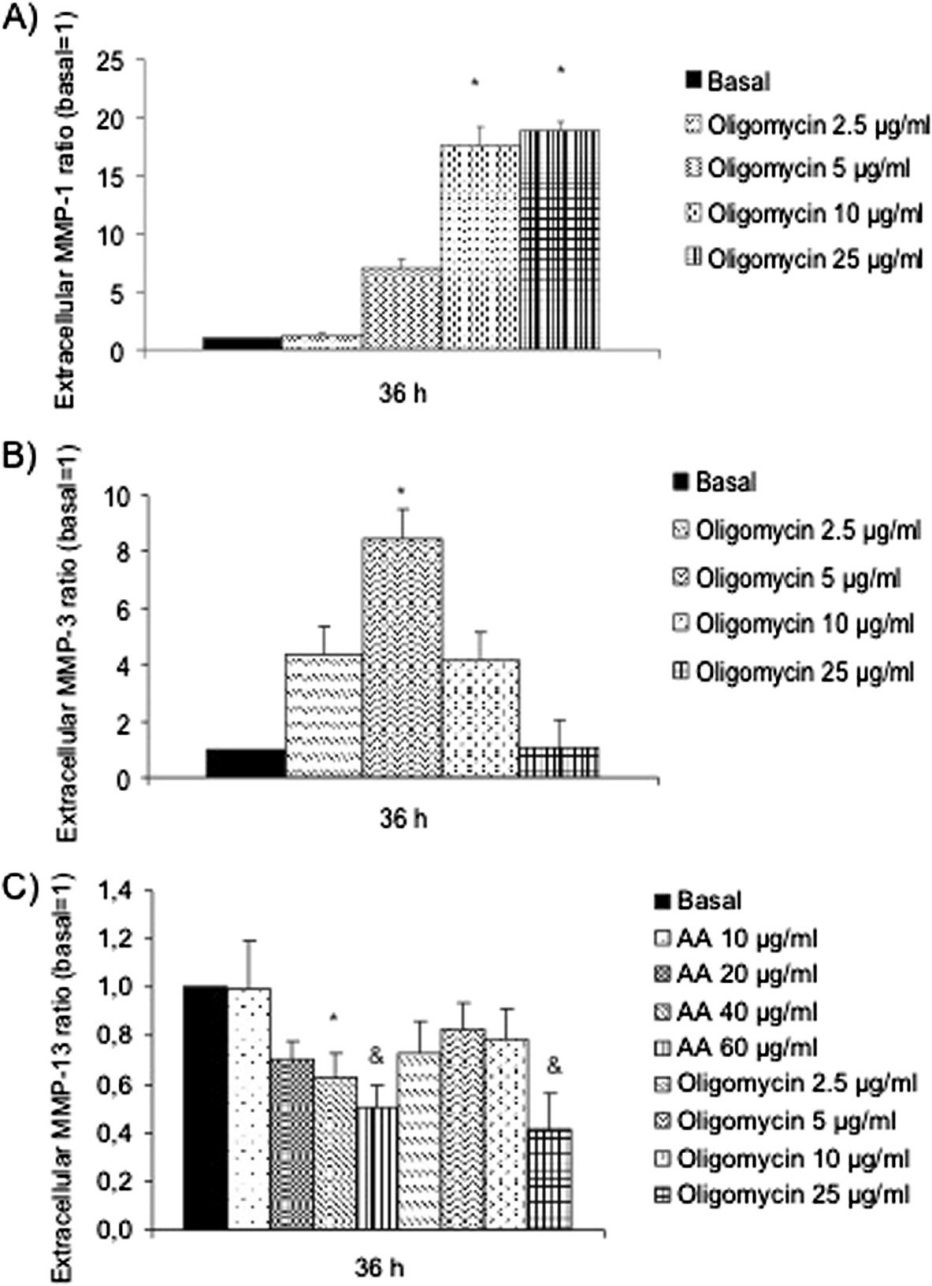
**Levels of MMPs liberated by chondrocytes after MRC dysfunction.** Human chondrocytes were cultured in 6 well plates in basal conditions, with AA or Oligomycin during 36 h and MMP -1 **(A)**, MMP-3 **(B)** and MMP-13 **(C)** were evaluated in supernatants. Data were represented as ratio of liberated protein representing the media ± SE of 5 experiments in duplo (*P<0.05; &*P*<0.01 *vs*. basal=1).

MMPs after being liberated by cells degrade the ECM. We measured their abundance in cellular supernatants after inhibiting the activity of the complexes III and V. AA (10, 20, 40 and 60 μg/ml) or Oligomycin (2.5, 5, 10 and 25 μg/ml) were added to chondrocyte cultures for 36 h. After recovering the supernatants, ELISA was performed to measure the quantity that was liberated to the media. The results confirmed that MMP-1 also increased after Oligomycin treatment (Figure 5A) 17.52±10.07 with 10 μg/ml and 18.06±10.35 with 25 μg/ml vs. basal=1; n=5; *P<0.05). MMP-3 ELISA (Figure 5B) showed the protein levels increased after Oligomycin 5 μg/ml treatment (at 36 h, 8.49±4.32 vs. basal=1; n=5; *P<0.05). MMP-13 (Figure 5C) decreased after the MRC inhibition as we already observed by western blot to 0.63±0.13, 0.50±0.13 with AA 40 and 60 μg/ml; and 0.41±0.14 with Oligomycin 25 μg/ml vs. basal=1 (n=5; * *P*<0.05; &*P*<0.01).

### Studies of MMPs in tissue after the induction of the MRC dysfunction

**Figure 6:**
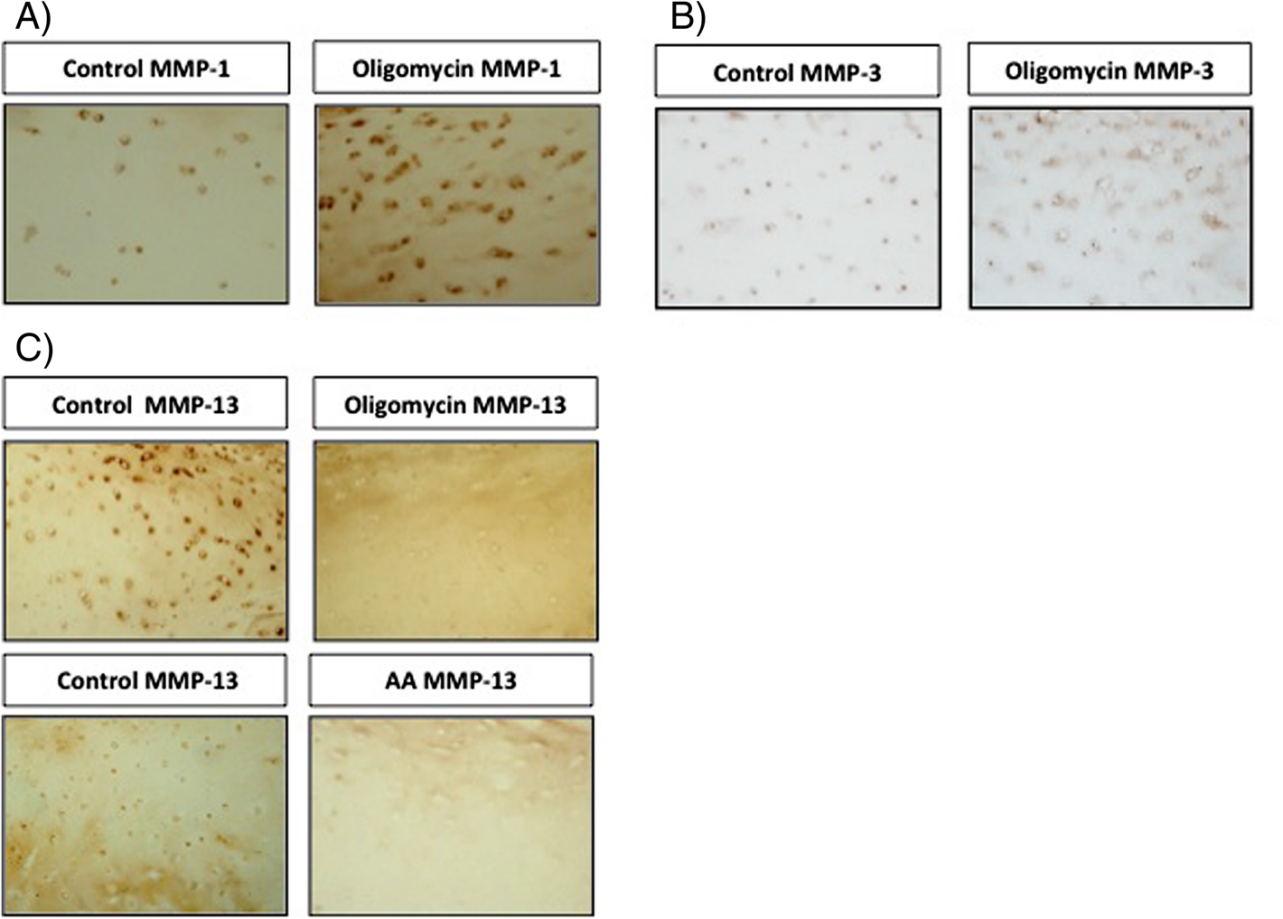
**MMPs evaluation in cartilage explants after MRC inhibition.** Cartilage explants from human normal donors were treated with Oligomycin 5 μg/mL or AA 20 μg/mL for 72 h and MMP-1 **(A)**, -3 **(B)** and -13 **(C)** were evaluated by immunohistochemistry. Example of a representative experiment of 3.

We tested whether the results were reproduced at tissue level. We stimulated cartilage explants with AA 20 μg/ml or Oligomycin 5 μg/ml during 72 h. Positivity for MMP-1 and -3 increased after Oligomycin treatment (Figure 6A, 6B) like we observed at cellular level. MMP-13, decreased after AA and Oligomycin (6C) stimulation.

### Proteoglycan detection after MRC dysfunction

**Figure 7:**
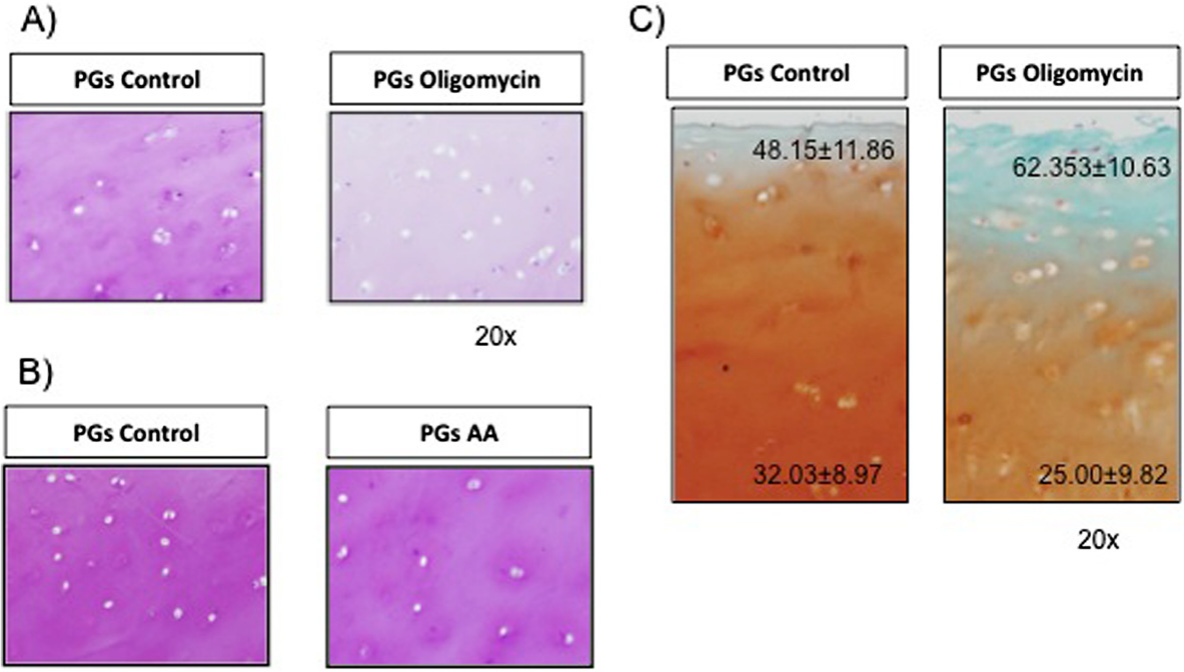
**Proteoglycan presence in tissues treated with MRC inhibitors.** Cartilage explants were treated with Oligomycin 5 μg/mL **(A)** or AA 20 μg/mL **(B)** for 72 h and proteoglycanes were stained with touidine blue. Safranine/fast-green was also used to detect proteoglycans with Oligomycin **(C)**. The figure represents an experiment of 3.

MMPs degrade the cartilage affecting the proteoglycan integrity and quantity. We evaluated the levels in tissue sections with toulidin blue staining. Results showed that Oligomycin 5 μg/ml produced a decrease in the proteoglycan quantity (Figure 7A). Figure 7B shows tissue explants treated with the complex III inhibitor (AA 20 μg/ml) for 72 h. Results indicated that the levels of proteoglycans did not change with AA. However, Oligomycin 5 μg/mL reduced proteoglycan quantities as safranin staining shows in Figure 7C (25.00±9.82 vs. basal=32.03±8.97).

## Discussion

The mitochondrion is a critic sensor of cell functionality and survival [23,24]. Several works support the idea that mitochondrion is linked to ageing and that mtDNA mutations due to oxidative stress contribute to cell death [25,26]. There is a direct relation between MRC function, energy production and ROS levels. The low activity of the MRC complexes III and V in brain tissues is related to Down syndrome and Alzheimer [27]. In other diseases like Parkinson, deficiencies in complex I have been found. In relation to this, different animal models of Parkinson have been developed in mice with rotenone [28,29]. In addition, a deficiency in complexes I, III and complex IV, could contribute to the development of Hungtington disease and Frederick ataxia [30,31].

OA and the diseases associated to ageing processes are known by the active process of cartilage degradation and chondrocyte death [32]. Considering the cartilage structure, the mitochondria should not have an important role since this is an avascular tissue [33]. Synovial membrane and subchondral bone are the tissues that feed the cartilage. However 25% of the ATP generated in the cartilage is produced from the OXPHOS system [34,35]. The main source of O_2_ is the synovial membrane and mitochondria can use this O_2_ in the aerobic respiration [36]. The role of mitochondria in OA has been extensively studied [35,37]. The activity of the MRC complexes II and III of OA chondrocytes is decreased respect healthy chondrocytes. Besides, cytokines and other OA mediators can regulate the activity of the MRC. For instance, TNF-α and IL-1β inhibit the activity of the MRC complex I [38]. This fact produces a decrease in the ATP levels and in the potential of the mitochondrial membrane. The last consequences are the reduction of the proteoglycan levels and cartilage functionality. Another mediator related to inflammatory processes is NO which has affinity for the mitochondrial complex IV reducing the ATP levels and the cell viability [9,39]. Chondrocytes stimulated with the NO donor 3-morpholinosydnonimine (SIN-1) increase their apoptotic population due to the caspase activity [40]. However the role of the MRC in the MMP regulation has never been studied.

The use of mitochondrial inhibitors has been extended to simulate different disorders [41,42]. Inhibitors of the complex II are used to simulate the Huntington disease [43,44]. AA and Oligomycin have been used also in the study of OA. A lower synthesis of collagen, a reduction of the action of (TGFβ) and ATP levels can be observed after altering the MRC [45]. Our group has described how AA and Oligomycin increased ROS production, Ca^2+^ transport and NF-κB activation with the activation of COX-2 and PGE_2_ production [13].

In our work we have evaluated the relation between the MRC activity and the ECM remodelling observing that the dysfunction of the MRC complexes III and V induced the MMP regulation. MMPs -1 and -3 were up-regulated at mRNA, intracellular protein and liberated protein levels after Oligomycin treatment. MMP-13 reduced its expression after the inhibition of complex III and V. Although MMP-13 is clue in the first phases of the OA, MMP-1 and -3 are more important in OA late phase [46]. MMP-13 can be regulated by proteins and factors and MMPs -1 and -3 would not be regulated by these members. For example p38 and Runx2 regulate in a positive way MMP-13 expression. Thus other authors have shown that the inhibition of p38 phosphorylation in chondrosarcome cells can be effective downregulating MMP-13 [47]. Mendes *et al*., observed that H_2_O_2_ generated by IL-1β, is an inductor of AP-1 factor which regulate many MMPs (46). Considering that mitochondria is an important source of ROS we could think that the MRC dysfunction would regulate the levels of MMP. In addition a production of ROS under the effect of AA and Oligomycin has been described [45]. Interestingly in a previous work we have demonstrated that Oligomycin and Antimycin can produce ROS in chondrocytes in culture and that this effect can be reversed after adding radical scavengers to the media [13].

Other authors have shown that ROS produced by the mitochondria are key regulators of MMP production and that these interactions are clearly important in disease pathologies [48,49].

Our results at cellular level were reproduced in tissue explants were we observed an increase in MMPs -1 and -3 after the inhibition of the complex V and a reduction of MMP-13 after the dysfunction of the complexes III and V. In addition, Oligomycin reduced the proteoglycan levels contributing to the destruction of the tissue. Other authors have also correlated the role of Oligomycin with the tissue integrity and proteoglycan synthesis [45]. We have observed different responses at mRNA, intracellular and extracellular level depending on the dosage of the inhibitor we have used. One hypothesis to explain this is that there is not always a linear relationship between mRNA, intracellular protein and extracellular protein expression. Future experiments inhibiting the activity of complex III and V with siRNA will be performed in order to understand the relation between levels of MRC activity and MMP expression/production.

Other authors have demonstrated that the regulation of the MRC function can directly affect the composition of the ECM [50]. For instance, De Cavanagh et al. have hypothesized that the depression of mitochondrial energy metabolism by ANG II is preceded by ANG II-induced integrin signaling and this produces the consequent derangement of the cytoskeletal filament network and/or ECM organization. However, to our knowledge, this is the first study that correlates the MRC dysfunction with the MMP production and the remodelling of the ECM in human cartilage. The pathways that regulate these processes should be studied in order to reveal the process of cartilage destruction in the OA.

## Conclusions

These results reveal that the MRC dysfunction modulates the MMPs expression in human normal chondrocytes demonstrating its role in the regulation of the cartilage destruction.

## Competing interests

In the past five years, we have not received any reimbursements, fees, funding, or salary from any organization that may in any way gain or lose financially from the publication of this manuscript. There is no organization financing this manuscript (including the article processing charge). We do not hold any stocks or shares in any organization that may in any way gain or lose financially from the publication of this manuscript. We do not currently hold any patents nor are we applying for any patents relating to the content of the manuscript.

We have not received reimbursements, fees, funding, or salary from any organization that holds or has applied for patents relating to the content of the manuscript.

There are no non-financial competing interests (political, personal, religious, ideological, academic, intellectual, commercial or any other) to declare in relation to this manuscript.

## Authors’ contributions

BCP carried out in the Western blot experiments as well as the Liberation of MMPs and its detection in tissue and the Proteoglycan studies. She has also performed the statistical analysis and drafted the manuscript. IRP carried out the mRNA expression studies. NO and CFL were involvement in the diagnostic of patients with OA as well as the procurement of the Cartilage samples. FJB conceived the idea of the study, and participated in its design and coordination. All authors read and approved the final manuscript.

## Pre-publication history

The pre-publication history for this paper can be accessed here:

http://www.biomedcentral.com/1471-2474/14/235/prepub
